# A Scalable Approach to Independent Vector Analysis by Shared Subspace Separation for Multi-Subject fMRI Analysis

**DOI:** 10.3390/s23115333

**Published:** 2023-06-05

**Authors:** Mingyu Sun, Ben Gabrielson, Mohammad Abu Baker Siddique Akhonda, Hanlu Yang, Francisco Laport, Vince Calhoun, Tülay Adali

**Affiliations:** 1 Department of Computer Science and Electrical Engineering, University of Maryland Baltimore County, Baltimore, MD 21250, USA; bengabr1@umbc.edu (B.G.); mo32@umbc.edu (M.A.B.S.A.); hyang3@umbc.edu (H.Y.); francisco.laport@udc.es (F.L.); 2 CITIC Research Center, University of A Coruña, 15008 A Coruña, Spain; 3 Tri-Institutional Center for Translational Research in Neuroimaging and Data Science (TReNDS), Georgia State University, Georgia Institute of Technology, and Emory University, Atlanta, GA 30303, USA; vcalhoun@gsu.edu

**Keywords:** independent vector analysis, MCCA, JBSS, subspace analysis, multi-subject medical imaging data, functional magnetic resonance imaging

## Abstract

Joint blind source separation (JBSS) has wide applications in modeling latent structures across multiple related datasets. However, JBSS is computationally prohibitive with high-dimensional data, limiting the number of datasets that can be included in a tractable analysis. Furthermore, JBSS may not be effective if the data’s true latent dimensionality is not adequately modeled, where severe overparameterization may lead to poor separation and time performance. In this paper, we propose a scalable JBSS method by modeling and separating the “shared” subspace from the data. The shared subspace is defined as the subset of latent sources that exists across all datasets, represented by groups of sources that collectively form a low-rank structure. Our method first provides the efficient initialization of the independent vector analysis (IVA) with a multivariate Gaussian source prior (IVA-G) specifically designed to estimate the shared sources. Estimated sources are then evaluated regarding whether they are shared, upon which further JBSS is applied separately to the shared and non-shared sources. This provides an effective means to reduce the dimensionality of the problem, improving analyses with larger numbers of datasets. We apply our method to resting-state fMRI datasets, demonstrating that our method can achieve an excellent estimation performance with significantly reduced computational costs.

## 1. Introduction

Data-driven methods that can produce informative features from the data while requiring very few assumptions are of great importance to the study of large datasets. Blind source separation (BSS) is a particular class of data-driven methods that separates latent sources from observed signals, and as such, BSS has demonstrated its usefulness across a wide range of applications [1,2,3]. Many applications involve multiple inherently related datasets, where it is reasonable to assume that latent sources within one dataset are related to some corresponding latent sources in one or more other datasets. This is especially prevalent in datasets acquired by functional magnetic resonance imaging (fMRI) [4,5], a standard neuroimaging tool for measuring brain function and activity due to its non-invasive nature and high resolution. In the study of multi-subject fMRI data, similar and consistent responses across multiple different subjects are indicative of shared functional network activities across those subjects. To fully leverage the joint information existing across the subjects’ datasets, data-driven methods need to model the latent dependencies across the datasets through a *joint* analysis. Joint blind source separation (JBSS) generalizes BSS to more than one dataset and is designed to model and exploit the dependence across datasets through a superior joint analysis [3,6,7,8,9]. JBSS methods operate by grouping sources that are dependent across the datasets, upon which JBSS methods typically maximize the dependence within each group (thus maximizing the dependence across the datasets). These groups are sometimes given the name “source component vector” (SCV) [3,6,7], and for the remainder of the paper, we refer to these groups as SCVs. Exploiting the dependence across datasets leads JBSS methods to achieve significantly more powerful estimators, capable of a superior source estimation performance and allowing them to better reveal the latent dependencies across the datasets [3,6,7,10].

One of the original methods for JBSS is the canonical correlation analysis (CCA), which transforms two datasets into two corresponding sets of variables known as “canonical variates” (CVs) that are maximally correlated across the two datasets [8,9,11]. A group of two variables that are maximally correlated with each other (one from each dataset) is functionally equivalent to an SCV; however, the CCA is only designed to analyze two datasets at a time. The multi-set canonical correlation analysis (MCCA) thus generalizes the CCA to two or more datasets [9,11,12] and similarly operates by transforming the data into variables that are maximally correlated across the datasets. Different MCCA algorithms maximize different empirical measures of “correlatedness” recorded from the covariance matrices of the SCVs. Of these algorithms, the most direct generalization of the CCA is the MCCA, which maximizes the sum of the correlations within an SCV covariance matrix (SUMCORR). The SUMCORR is notable in that it has been shown to effectively model each SCV by a rank 1 covariance [12]. This model is useful when SCVs can be regarded as repeats of a single “shared” source (i.e., a source that exists across all datasets), and thus, the MCCA SUMCORR is well-suited for estimating these corresponding “shared SCVs”. Another commonly used JBSS method is group-ICA [13,14,15,16], which, like the MCCA SUMCORR, assumes that all datasets in the joint analysis share the same sources at the group level. The MCCA SUMCORR and group-ICA both have simple implementations that are computationally well-suited to analyzing a large number of datasets; however, the assumption that all SCVs are “shared” effectively limits the performance when greater variability exists across the datasets (among sources within each SCV) [17].

When the assumption that sources are shared across subjects is not true, JBSS methods that are well-suited for modeling variability, such as the independent vector analysis (IVA), have greater success in data analysis [17]. The IVA operates by maximizing independence among SCVs, analogous to how the ICA maximizes independence among sources within a single dataset. By maximizing independence among SCVs, the IVA simultaneously maximizes independence among sources within each dataset *and* maximizes dependence within each SCV. Unlike other JBSS methods, such as the MCCA SUMCORR and group-ICA, the IVA does not operate under the assumption that all SCVs are shared SCVs; thus, the IVA provides a superior performance compared to other methods when greater variability exists across the datasets [17,18]. However, the performance of the IVA may be worse in the presence of all shared SCVs compared with methods such as the MCCA SUMCORR and group-ICA. This is, in part, due to most IVA algorithms parameterizing each SCV by a *K* dimensional multivariate distribution (with *K* being the number of datasets), whereas shared SCVs lie on a low-dimensional subspace and, thus, are at high risk for overparameterization. As a result, the performance of the IVA can be significantly degraded by the presence of shared SCVs, in terms of both separation performance and running time. Furthermore, IVA algorithms are burdened by higher computational complexity compared with methods such as the MCCA and group-ICA. As the IVA can be considered one of the most powerful and flexible methods for BSS [3,6,7,10,15,18,19], there exists a need for more efficient variants of IVA that can also perform well with shared SCVs.

For several types of related datasets encountered in JBSS, especially in the case of fMRI data, we may expect that many of the SCVs can be modeled reasonably as shared SCVs, while the remaining SCVs demonstrate significant variability across the datasets. For the remainder of the paper, we refer to the collection of latent SCVs that are shared as the “shared subspace” of the data and the remaining SCVs as the “non-shared subspace”. Tools for identifying the shared and non-shared subspaces have demonstrated their usefulness in fMRI analysis [4,5,20], allowing for the identification of shared functional network activations, in addition to those that may exhibit more variability across the datasets. The former can be useful for identifying activations that are consistently present across all subjects, whereas the latter can be useful for its discriminatory ability in further analysis (e.g., for diagnosing or classifying subjects). While MCCA and group-ICA methods typically perform better on data with shared SCVs, and the IVA typically performs better on data with non-shared SCVs, there exists a need for JBSS methods that are well-suited to the general case where data have both shared and non-shared SCVs and can explicitly exploit this information in the analysis.

To resolve the aforementioned issues, in this paper, we propose a method known as the independent vector analysis by shared subspace separation (IVA-S3). The proposed algorithm, IVA-S3, is designed to overcome some of the key issues with the IVA regarding both the computational complexity and performance in shared subspaces. The IVA-S3 models the shared subspaces within the data by utilizing the MCCA SUMCORR as a preprocessing step, thus allowing shared SCVs to be estimated prior to the IVA step at a level beyond the capabilities of conventional IVA methods. Once shared SCVs have been identified by a measure of “correlatedness” within the covariance matrices of the SCVs, the shared SCVs are separated from the remaining non-shared SCVs, and the IVA is performed on the shared and non-shared subspaces separately. This provides further refinement of these subspaces’ estimations while also considerably reducing the dimensionality of the data within the IVA step, leading to a significantly lower computational complexity. We compare the results of our proposed method with the group-ICA results, demonstrating the reliability of the estimation of SCVs with a sizeable dataset in addition to the outperformance of group-ICA in terms of multiple key performance metrics evaluated, including mutual information across sources, power ratios, and t-maps.

The remainder of this paper is organized as follows: Section 2 formulates the JBSS problem. Section 3 introduces the MCCA and IVA methods for performing JBSS. Section 4 presents the steps of the IVA-S3 method, including the MCCA SUMCORR preprocessing step, the identification of the shared subspace from the MCCA SUMCORR solution, and how IVA is performed separately on the shared and non-shared subspaces. Section 5 demonstrates the performance of the IVA-S3 in the context of simulated results as well as results obtained from real fMRI data. Section 6 concludes the paper with takeaways about the proposed method in addition to potential future directions.

## 2. JBSS Problem Formulation

We first mathematically formulate the general JBSS problem. We observe *K* datasets $x^{\left[k\right]}\left(t\right)\in R^{N}$ over *T* samples (t=1,…,T), and each dataset $x^{\left[k\right]}\left(t\right)$ = $[x_{1}^{\left[k\right]}\left(t\right)$, …, $x_{N}^{\left[k\right]}{\left(t\right)]}^{⊤}\in R^{N}$ is modeled as a linear mixture of *N* latent source signals $s^{\left[k\right]}\left(t\right)$ = $[s_{1}^{\left[k\right]}\left(t\right)$, …, $s_{N}^{\left[k\right]}{\left(t\right)]}^{⊤}$$\in R^{N},1\leq k\leq K$. The generative model is

(1) $$x^{\left[k\right]}\left(t\right)=A^{\left[k\right]}\;s^{\left[k\right]}\left(t\right),$$

where $A^{\left[k\right]}\in R^{N\times N}$ is an invertible mixing matrix. To estimate the latent sources within the *K* datasets, JBSS estimates *K* demixing matrices $W^{\left[k\right]}$$\in R^{N\times N}$ that demix each dataset into its corresponding estimated sources $y^{\left[k\right]}\left(t\right)$ = $[y_{1}^{\left[k\right]}\left(t\right)$, …, $y_{N}^{\left[k\right]}{\left(t\right)]}^{⊤}\in R^{N}$ where $y^{\left[k\right]}\left(t\right)=W^{\left[k\right]}\;x^{\left[k\right]}\left(t\right)$. The mixing matrices $A^{\left[k\right]}$ (or the demixing matrices $W^{\left[k\right]}$) can be distinct for different datasets, and they are not assumed to be related.

As the datasets are observed over *T* samples of data (t=1,…,T), the datasets are, in practice, represented by the matrices $X^{\left[k\right]}$ = [$x_{1}^{\left[k\right]}$, …, $x_{N}^{\left[k\right]}{]}^{⊤}$$\in R^{N\times T}$, and the generative model shown in Equation (1) is given as $X^{\left[k\right]}$ = $A^{\left[k\right]}$$S^{\left[k\right]}$, with latent sources given by $S^{\left[k\right]}$ = [$s_{1}^{\left[k\right]}$, …, $s_{N}^{\left[k\right]}{]}^{⊤}$$\in R^{N\times T}$ and estimated sources given by $Y^{\left[k\right]}$ = $W^{\left[k\right]}$ $X^{\left[k\right]}$ = [$y_{1}^{\left[k\right]}$, …, $y_{N}^{\left[k\right]}{]}^{⊤}$$\in R^{N\times T}$.

JBSS formulations assume a model where sources of the same index *n* are dependent across the *K* datasets, forming *N* sets of *K* sources. In IVA terminology, each of these groups of sources is known as a “source component vector” (SCV), and all JBSS methods fundamentally operate by either maximizing dependence within SCVs and/or minimizing dependence among the different SCVs. Each of the *N* latent SCVs is denoted by the vector $s_{n}$ = $[s_{n}^{\left[1\right]}$, …, $s_{n}^{\left[K\right]}{]}^{⊤}\in R^{K}$, which is estimated by the corresponding JBSS-estimated SCVs $y_{n}$ = $[y_{n}^{\left[1\right]}$, …, $y_{n}^{\left[K\right]}{]}^{⊤}\in R^{K}$. Over *T* samples of data, the latent SCVs are denoted by the matrices $S_{n}$ = $[s_{n}^{\left[1\right]}$, …, $s_{n}^{\left[K\right]}{]}^{⊤}\in R^{K\times T}$, which are estimated by $Y_{n}$ = $[y_{n}^{\left[1\right]}$, …, $y_{n}^{\left[K\right]}{]}^{⊤}\in R^{K\times T}$. In addition, it is conventional to standardize and whiten the data prior to JBSS, since the math involved in performing these methods becomes considerably simplified [1]. For the remainder of the paper, we assume that the sources and datasets are standardized, $∥w_{n}^{\left[k\right]}{∥}_{2}=1$ and the datasets are prewhitened, where ${\left(w_{n}^{\left[k\right]}\right)}^{⊤}$ is the *n*th row of demixing matrix $W^{\left[k\right]}$, and it is used to estimate the *n*th source within the *k*th dataset, given by $y_{n}^{\left[k\right]}\left(t\right)={\left(w_{n}^{\left[k\right]}\right)}^{⊤}\;x^{\left[k\right]}\left(t\right)$.

## 3. Methods for JBSS

### 3.1. MCCA

The MCCA is a multivariate statistical method that is used to find relationships between multiple sets of variables. The goal of the MCCA is to estimate demixing vectors $w_{n}^{\left[k\right]}$ corresponding to the SCV $y_{n}$ in order to maximize the correlatedness within that SCV (measured by the SCV’s covariance ${\hat{\Sigma }}_{y_{n}}$ = $\frac{1}{T-1}$$Y_{n}$$Y_{n}^{⊤}$$\in R^{K\times K}$). There are five different versions of the MCCA, each employing a different cost function over a different empirical measure of correlatedness in ${\hat{\Sigma }}_{y_{n}}$. One of the most commonly used variants of the MCCA is the MCCA SUMCORR, whose objective function is to maximize the sum of correlations within an SCV’s covariance. It has been previously shown [12] that the SUMCORR procedure inherently assumes a rank 1 model for each SCV, i.e., a single source that is “shared” across the *K* datasets.

(2) $$S_{n}=1_{n}v_{n}^{⊤}+Z_{n},\;1\leq n\leq N,$$

where $v_{n}$ $\in R^{T}$ is the shared source existing across all datasets, $1_{n}$$\in R^{K}$ is a vector of 1s scaled by some scalar value, and $Z_{n}$$\in R^{K\times T}$ is some additive noise with a diagonal covariance matrix. The SUMCORR has previously demonstrated many useful decompositions over fMRI datasets [21], implying that many latent fMRI SCVs may be well-suited to this “shared source” model.

The implicit nature of the SUMCORR model makes it well-suited to the estimation of shared SCVs; however, the model and performance of the SUMCORR may be inadequate when SCVs are not shared (i.e., they demonstrate significant variability within themselves). While the rank 1 model assumed by the SUMCORR is expected to poorly estimate non-shared SCVs, the SUMCORR retains the ability to estimate shared SCVs in the presence of non-shared SCVs (e.g., when the data are a mixture of some shared and some non-shared). One approach to improving the performance of JBSS over that of SUMCORR [21] operates by applying a joint diagonalization procedure to refine the SUMCORR-estimated sources. Nevertheless this approach still fundamentally assumes the effective rank 1 SCV model for all SCVs, and thus is limited when more variability exists within the data.

One of the primary benefits that SUMCORR has over other JBSS algorithms is its simplicity and low computational complexity. The SUMCORR solution is solved by simply concatenating the datasets into a single tall matrix X = $[{\left(X^{\left[1\right]}\right)}^{⊤}$, …, ${\left(X^{\left[K\right]}\right)}^{⊤}{]}^{⊤}$$\in R^{NK\times T}$, computing its sample covariance ${\hat{C}}_{x}$ = $\frac{1}{T-1}$X$X^{⊤}$$\in R^{NK\times NK}$, and computing the *N* principal eigenvectors of ${\hat{C}}_{x}$. It is straightforward to show that the *n*th principal eigenvector of ${\hat{C}}_{x}$, denoted by ${\hat{v}}_{x}$${}_{n}$$\in R^{NK}$, is the concatenation of scaled SUMCORR demixing vectors of the *n*th SCV: ${\hat{v}}_{x}$${}_{n}$ = [${\left(w_{n}^{\left[1\right]}\right)}^{⊤}$, *…*, ${\left(w_{n}^{\left[K\right]}\right)}^{⊤}$]${}^{⊤}$. This puts the SUMCORR among the most computationally efficient of all JBSS algorithms, with an asymptotic computational complexity of O( $N^{3}K^{3}$ ).

### 3.2. IVA

Assuming that the latent SCVs are independent, the mutual information between the SCVs is a natural cost of performing JBSS. Analogous to the ICA, which maximizes the independence among the source components, the IVA operates by maximizing the independence among the *N* SCVs, thus minimizing the mutual information between the estimated SCVs $y_{n}$. The IVA models each SCV with a multivariate PDF, allowing the IVA to fully exploit the statistical dependence across datasets via the SCVs. The mutual information cost is given by

(3) $$\begin{array}{c} I_{IVA}\left(W\right)≜\sum_{n=1}^{N}H\left(y_{n}\right)-\sum_{k=1}^{K}\log\left|\det\left(W^{\left[k\right]}\right)\right| \end{array},$$

where $y_{n}$ is the *n*th SCV, and $H(y_{n})$ denotes the entropy of the source $y_{n}$ defined on the PDF $p(y_{n})$. Noting $H\left(y_{n}\right)=\sum_{k=1}^{K}H\left(y_{n}^{\left[k\right]}\right)-I\left(y_{n}\right)$, Equation (3) can be written as

(4) $$I_{IVA}\left(W\right)≜\sum_{n=1}^{N}\left(\sum_{k=1}^{K}H\left(y_{n}^{\left[k\right]}\right)-I\left(y_{n}\right)\right)-\sum_{n=k}^{K}\log\left|\det\left(W^{\left[k\right]}\right)\right|.$$

This reveals that minimizing the mutual information across SCVs has the effect of simultaneously minimizing the entropy of all components (which can be shown from ICA formulations as minimizing the dependence across SCVs) and maximizing the mutual information within SCVs (maximizing dependence within SCVs). As a result, it is convenient to view the minimizing Equation (4) as both minimizing the dependence among SCVs and maximizing the dependence within each SCV.

If all *N* SCVs are modeled as multivariate Gaussian-distributed, i.e., $y_{n}∼N\left(0,\Sigma_{y_{n}}\right)$, then the IVA exclusively exploits second-order statistics (i.e., correlations), and thus effectively minimizes the correlations among SCVs while maximizing the correlations within each SCV. This version of the IVA is known as the independent vector analysis with a multivariate Gaussian source prior (IVA-G) [3,6,7]. The cost function of the IVA-G is derived by assuming the multivariate Gaussian PDF as being the distribution of each SCV, given by $p_{n}\left(y_{n}|\Sigma_{y_{n}}\right)=\frac{1}{{\left(2\pi \right)}^{\frac{K}{2}}\det{\left(\Sigma_{y_{n}}\right)}^{\frac{1}{2}}}\exp\left(-\frac{1}{2}y_{n}^{T}\Sigma_{y_{n}}^{-1}y_{n}\right)$, where $\Sigma_{y_{n}}\in R^{K\times K}$, where $\Sigma_{y_{n}}$ is the covariance of the *n*th estimated SCV, and in practice, this covariance is estimated over *T* samples by the unbiased estimator ${\hat{\Sigma }}_{y_{n}}=\frac{1}{T-1}Y_{n}Y_{n}^{T}$. The IVA-G cost is given by

(5) $$\begin{array}{cc} C_{IVA-G}\left(W\right) & =\frac{NK\log(2\pi e)}{2}+\frac{1}{2}\sum_{n=1}^{N}\log|\det\left({\hat{\Sigma }}_{y_{n}}\right)|-\sum_{k=1}^{K}\log\left|\det\left(W^{\left[k\right]}\right)\right|\\ \; & =\frac{NK\log(2\pi e)}{2}+\frac{1}{2}\log\left(\prod_{n=1}^{N}\prod_{k=1}^{K}\lambda_{n}^{\left[k\right]}\right)-\sum_{k=1}^{K}\log\left|\det\left(W^{\left[k\right]}\right)\right|, \end{array}$$

where $\lambda_{n}^{\left[k\right]}$ is the *k*th largest eigenvalue of the covariance ${\hat{\Sigma }}_{y_{n}}$. Regarding the 2nd term of Equation (5), it is important to note that determinant-based costs are disproportionately affected by the smallest eigenvalues present. In practice, the smallest eigenvalues are most likely to describe the “noise” space in the data, in contrast to the largest eigenvalues, which describe the “signal” space in the data. Because of this inherent sensitivity to smaller eigenvalues, the IVA-G is especially prone to overfitting if the dimension of the “signal” space is small, i.e., if the SCV lies on a subspace with a much lower dimensionality than its dimension *K*. However, for the case with shared SCVs, we expect a single dominant “signal” eigenvalue and K−1 “noise” eigenvalues. As a result, the IVA-G is well-suited to the estimation of SCVs with significant levels of variability, that is, high numbers of “signal” eigenvalues, but it may significantly overparameterize, and thus poorly estimate, shared SCVs.

In addition, as the IVA-G only exploits the linear dependence between the sources, the IVA-G only needs to calculate a single covariance matrix over the data (the same matrix ${\hat{C}}_{x}$ calculated in SUMCORR), and thus, the asymptotic computational complexity is essentially independent of the number of samples *T* (instead, it is dominated by the number of SCVs *N* and the number of datasets *K*). This leads the IVA-G to be among the most computationally efficient IVA algorithms. However, unlike the SUMCORR, the IVA-G requires an iterative optimization procedure with significant computational complexity at each iteration. If the IVA-G algorithm requires *q* iterations to converge, it is straightforward to show that the IVA-G has a total complexity of O( $q(N^{4}K+N^{3}K^{2}+NK^{4})$ ). While this complexity is prohibitive for very large numbers of SCVs *N* or datasets *K*, the complexity can be significantly reduced if either *N* or *K* can be reduced within the JBSS procedure.

As the IVA-G models SCVs as multivariate Gaussian, the IVA-G only exploits second-order statistical relationships without exploiting any higher-order statistics (HOS). However, the multivariate Laplacian distribution appears to be a better model match for fMRI data [22,23]; thus, it is generally desirable to exploit HOS when analyzing these fMRI datasets. In this context, a relevant IVA algorithm known as IVA-L-SOS [19,22] is able to exploit second-order statistics within the data, while also modeling the SCVs by a multivariate Laplacian distribution. Because of this additional ability to exploit the Laplacian nature of the latent fMRI sources, the IVA-L-SOS has been proven to be very powerful for extracting interpretable source components for fMRI analysis [19,22]. However, despite the possibly improved estimation performance with the IVA-L-SOS, the computational complexity of the IVA-L-SOS is much higher than that of the IVA-G due to the need to apply non-linear functions on each sample at each iteration. This leads to a high per-sample complexity, effectively limiting the practical use of the IVA-L-SOS when applied to a very large number of fMRI datasets.

Considering the advantages and limitations of these various JBSS algorithms, in the next section, we explain our proposed method (IVA-S3) in relation to these previous methods.

## 4. IVA by Shared Subspace Separation

### 4.1. IVA with SUMCORR Initialization

Given that JBSS methods such as the IVA are frequently applied to fMRI datasets, it is prudent to use methods that specifically exploit the inherent properties of fMRI data to improve the overall decomposition. Due to the inherent similarity in functional network activity across different subjects, it is reasonable to assume that many components are shared among the subjects; thus, many SCVs can be well-modeled as shared SCVs. It is also reasonable to assume that some components may also not be shared, and thus, the best JBSS approaches are those that can estimate both shared and non-shared SCVs.

Despite the fact that the IVA-G can encounter estimation issues with shared SCVs, the IVA-G may actually estimate the shared SCVs well if the IVA-G is given a good initialization process, specifically one that also provides good initialization to shared SCVs. As the SUMCORR is well-suited to the estimation of shared SCVs, and as the SUMCORR is among the most efficient methods for JBSS, the SUMCORR is an ideal candidate for providing a guiding initialization to the IVA-G. In this sense, not only does initializing with the SUMCORR help to adequately estimate the shared SCVs, it also helps to significantly reduce the wall time of the JBSS by providing a fast initialization process that is reasonably close to the IVA-G solution. Aside from these benefits, the SUMCORR is superior to other random initialization-based approaches, such as IVA-G with a random initialization, that aim to find the “most representative run” out of multiple runs of the IVA-G [24,25], as the SUMCORR will consistently give the same results every time, since it is an analytic solution. This not only facilitates the interpretability of the result but makes JBSS feasible for many applications with high-dimensional data.

### 4.2. Shared Subspace Identification and Separation

We propose a simple method for determining whether an SCV $Y_{n}$ is shared by analyzing the eigenvalues of the SCV’s covariance matrix, ${\hat{\Sigma }}_{y_{n}}$. The eigenspectra of a shared SCV’s covariance ${\hat{\Sigma }}_{y_{n}}$ is characterized by a single dominant eigenvalue $\lambda_{n1}$ corresponding to the shared source, with $\lambda_{n1}\approx K$ (since ${\hat{\Sigma }}_{y_{n}}$ is also a correlation matrix, and thus, the *K* eigenvalues sum to *K*), while the remaining K−1 eigenvalues are close to zero and correspond to noise. This can also be understood from the perspective of the principal component analysis (PCA): with shared SCVs, one single principal component (PC) effectively represents all sources within the SCV (this PC effectively *is* the shared source), and thus, the corresponding largest eigenvalue of the covariance matrix explains the overwhelming majority of the variance. In contrast, the variability in non-shared SCVs is insufficiently described by a single PC, in which case the covariance’s largest eigenvalue $\lambda_{n1}$ is much closer to the second-largest eigenvalue $\lambda_{n2}.$ We define this difference $\lambda_{n1}-\lambda_{n2}$ as the “spectral gap” of the *n*th SCVs covariance, effectively one measure of how well an SCV is represented by a shared source. We note the way that we define the spectral gap here is distinct from the “spectral gap” in physics or graph theory. This measure of the spectral gap can be a useful measure for gauging the potential performance of both the MCCA SUMCORR and IVA-G: larger spectral gaps indicate a better match to the rank 1 assumption of the SUMCORR, in which case, the SUMCORR is expected to yield a better estimation performance for these SCVs, whereas smaller spectral gaps indicate SCVs that are better estimated by the IVA-G.

As the spectral gap can vary depending on the number of datasets *K*, we can improve this measure by normalizing it with respect to the largest eigenvalue and thus provide a more standardized measure that is not affected by *K*. We call the resulting measure the “spectral gap ratio”, which is defined as

(6) $$\sigma_{n}=\frac{\lambda_{n1}-\lambda_{n2}}{\lambda_{n1}}.$$

Larger values of this spectral gap ratio $\sigma_{n}$ are indicative of SCVs that are better modeled by a shared source. In our experience with handling fMRI datasets, 0.86 is a useful threshold for the spectral gap: SCVs with $\sigma_{n}>0.86$ are well-described by the shared SCV model, whereas SCVs with $\sigma_{n}\leq 0.86$ exhibit enough variability to be sufficiently estimated by the IVA-G alone. Thus, for the remainder of this work, we define all SCVs with $\sigma_{n}>0.86$ as shared SCVs but note that this threshold may be modified on a per-application basis (we use Δ to notate this threshold).

### 4.3. IVA-S3 Framework

**Figure 1:**
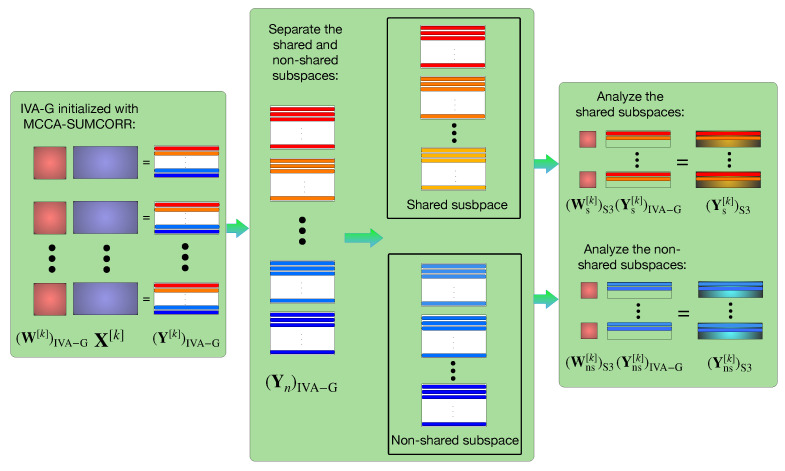
General framework of the IVA-S3: In the first stage, IVA-G with SUMCORR initialization is applied to the entire dataset; in the second stage, the shared subspaces are identified and separated from the non-shared subspaces; in the third stage, IVA is applied separately to the shared and non-shared subspaces.

The proposed method, IVA-S3, is summarized by the procedure outlined in Figure 1. First, the MCCA SUMCORR is performed on the data $X^{\left[k\right]}$ to provide an initialization procedure for the IVA-G that assists in estimating the shared SCVs beyond the capabilities of the IVA-G alone. Then, given the SUMCORR-initialized IVA-G solution ${\left(W^{\left[k\right]}\right)}_{\mathrm{IVA}-G}$ and ${\left(Y^{\left[k\right]}\right)}_{\mathrm{IVA}-G}={\left(W^{\left[k\right]}\right)}_{\mathrm{IVA}-G}X^{\left[k\right]}$, the performance of the decomposition is improved by separately analyzing the shared and non-shared subspaces. SCVs are labeled as shared or non-shared based on their corresponding spectral gap ratios $\sigma_{n}$, and the estimated sources for each dataset $Y^{\left[k\right]}\in R^{N\times T}$ are separated into that dataset’s corresponding shared sources ${\left(Y_{s}^{\left[k\right]}\right)}_{\mathrm{IVA}-G}\in R^{N_{s}\times T}$ and non-shared sources ${\left(Y_{\mathrm{ns}}^{\left[k\right]}\right)}_{\mathrm{IVA}-G}\in R^{N_{\mathrm{ns}}\times T}$, where $N_{s}$ is the number of shared SCVs, $N_{\mathrm{ns}}$ is the number of non-shared SCVs, and $N_{s}$ + $N_{\mathrm{ns}}$ = *N*. Then, with the shared and non-shared SCVs separated into two different groups, the IVA is applied separately to the shared and the non-shared SCVs, with ${\left(Y_{s}^{\left[k\right]}\right)}_{\mathrm{IVA}-G}$ and ${\left(Y_{\mathrm{ns}}^{\left[k\right]}\right)}_{\mathrm{IVA}-G}$ supplied as the datasets within the resulting decompositions, and both separate procedures initializing the demixing matrices with identity matrices (thus initializing with the sources estimated by the previous stage). Namely, we have ${\left(Y_{s}^{\left[k\right]}\right)}_{S3}={\left(W_{s}^{\left[k\right]}\right)}_{S3}{\left(Y_{s}^{\left[k\right]}\right)}_{\mathrm{IVA}-G}$ and ${\left(Y_{\mathrm{ns}}^{\left[k\right]}\right)}_{S3}={\left(W_{\mathrm{ns}}^{\left[k\right]}\right)}_{S3}{\left(Y_{\mathrm{ns}}^{\left[k\right]}\right)}_{\mathrm{IVA}-G}$ for the final stage of the IVA-S3, and ${\left(Y_{\mathrm{ns}}^{\left[k\right]}\right)}_{S3}$ and ${\left(Y_{\mathrm{ns}}^{\left[k\right]}\right)}_{S3}$ are our final estimations. These separate estimation procedures are not necessarily limited to the performance of the IVA-G on the separate subspaces, and other IVA algorithms that exploit more statistical properties of the data can be used instead (e.g., IVA algorithms such as the IVA-L-SOS that exploit the Laplacian nature of fMRI sources). This subspace separation procedure leads to reduced dimensions $N_{s}$ and $N_{\mathrm{ns}}$ in the resulting decompositions, ultimately resulting in a lower complexity and improvement of the wall time performance. Below, we also provide a pseudocode (Algorithm  1) that summarizes the IVA-S3 method:
**Algorithm 1** IVA-S3**Require:** datasets X = $[{\left(X^{\left[1\right]}\right)}^{⊤}$, …, ${\left(X^{\left[K\right]}\right)}^{⊤}{]}^{⊤}$$\in R^{NK\times T}$ $W_{\mathrm{init}}\leftarrow$ MCCA SUMCORR (X) $W_{\mathrm{IVA}-G}\leftarrow$ IVA-G ($X,W_{\mathrm{init}}$) ${\left(Y^{\left[k\right]}\right)}_{\mathrm{IVA}-G}\leftarrow$
${\left(W^{\left[k\right]}\right)}_{\mathrm{IVA}-G}X^{\left[k\right]}$ $y_{n}\leftarrow {\left(Y^{\left[k\right]}\right)}_{\mathrm{IVA}-G}$ **for** n=1:N 
**do**    $\sigma_{n}=\frac{\lambda_{n1}-\lambda_{n2}}{\lambda_{n1}}$    **if** $\sigma_{n}>\Delta$ **then**        Shared subspace ←$y_{n}$    **else**        Non-shared subspace ←$y_{n}$    **end if** **end for** ${\left(Y_{s}^{\left[k\right]}\right)}_{\mathrm{IVA}-G}\leftarrow$ shared subspace, ${\left(Y_{\mathrm{ns}}^{\left[k\right]}\right)}_{\mathrm{IVA}-G}\leftarrow$ non-shared subspace $W_{s}\leftarrow$ IVA (${\left(Y_{s}^{\left[k\right]}\right)}_{\mathrm{IVA}-G},I$), $W_{\mathrm{ns}}\leftarrow$ IVA (${\left(Y_{\mathrm{ns}}^{\left[k\right]}\right)}_{\mathrm{IVA}-G},I$) ${\left(Y_{s}^{\left[k\right]}\right)}_{S3}\leftarrow$${\left(W_{s}^{\left[k\right]}\right)}_{S3}{\left(Y_{s}^{\left[k\right]}\right)}_{\mathrm{IVA}-G}$, ${\left(Y_{\mathrm{ns}}^{\left[k\right]}\right)}_{S3}\leftarrow$${\left(W_{\mathrm{ns}}^{\left[k\right]}\right)}_{S3}{\left(Y_{\mathrm{ns}}^{\left[k\right]}\right)}_{\mathrm{IVA}-G}$

## 5. Results

### 5.1. Performance with Simulated Data

To evaluate the performance of our method and compare it with existing methods, we conducted a series of simulations. Our application focus was on fMRI data analysis, where many of the source components are likely to be shared among subjects. We simulated shared SCVs by defining their covariance matrices with an “effective rank” of 1, where the principal eigenvalue corresponds to the signal and the remaining K−1 eigenvalues correspond to the noise. The generative model of each SCV covariance is thus given by

(7) $$\Sigma_{s_{n}}=\mu_{n}1_{n}1_{n}^{⊤}+\eta I_{k}$$

where $\mu_{n}\in$ [0 1] defines the value taken by all off-diagonal entries in the covariance, $1_{n}$ is a vector of 1s, and $I_{k}$ is the identity matrix. To ensure that the covariance matrix was also a correlation matrix, we also set η within [0 1] such that $\mu_{n}+\eta =1$. In our simulations, we used equally spaced values of μ ranging from 0.80 to 0.50. In contrast to shared SCVs with “simple” covariances, we generated non-shared SCVs with more “complex” covariances, such that the non-shared covariances had an effective rank equal to *K* (“effective full rank”). To generate these more “complex” covariances, we generated covariances of the form $QQ^{⊤}$, where the matrix $Q\in R^{K\times K}$ is a random matrix with each row drawn from the standard Gaussian distribution and then row-normalized to the unit norm. As a result, the matrix $QQ^{⊤}$ became a random correlation matrix (with the values on the diagonal equal to 1 and the values on the diagonal being random values between −1 and 1).

Latent sources within fMRI data are generally described by the multivariate Laplacian distribution; thus, to include this within our simulations, we generated our SCVs using a multivariate generalized Gaussian distributed PDF model (MGGD) [26] with the distribution shape parameter β = 0.5. Once the SCVs $S_{n}$ had been generated, sources therein were distributed to their corresponding datasets $S^{\left[k\right]}$ and then mixed by mixing matrices $A^{\left[k\right]}$, with values in $A^{\left[k\right]}$ drawn from the standard Gaussian distribution. We generated a total of N=50 sources, with K=100 datasets and a sample size of T=20NK. All MGGD sources were normalized to a zero mean and unit variance; hence, the covariance and correlation coefficients coincided. We simulated three scenarios:

- All 50 SCVs are shared;
- All 50 SCVs are non-shared;
- Half (25) are shared, and half (25) are non-shared.

We compared four algorithms: group-ICA, SUMCORR, IVA-G, and IVA-S3 (with the IVA-S3 using the IVA-G in the final stage). As we knew the ground truth for the simulated data, we qualified the performance by joint-intersymbol interference (joint-ISI), which is defined as

(8) $${\mathrm{ISI}}_{J}\left(G^{\left[1\right]},G^{\left[2\right]},...,G^{\left[K\right]}\right)≜\mathrm{ISI}(\frac{1}{K}\sum_{k=1}^{K}|G^{\left[K\right]}\left|\right)$$

where

(9) $$\begin{array}{cc} \mathrm{ISI}(G)= & \frac{1}{2N(N-1)}(\sum_{n=1}^{N}\left(\frac{\sum_{m=1}^{N}\left|g_{nm}\right|}{\max_{p}\left|g_{np}\right|}-1\right)\\ \; & +\sum_{m=1}^{N}\left(\frac{\sum_{n=1}^{N}\left|g_{nm}\right|}{\max_{p}\left|g_{pm}\right|}-1\right)) \end{array}$$

where $G^{\left[k\right]}=W^{\left[k\right]}A^{\left[k\right]}$, with elements denoted as $g_{mn}$ [24,25]. The joint-ISI is a normalized performance measure with values between 0 and 1 that captures how close each $G^{\left[k\right]}=W^{\left[k\right]}A^{\left[k\right]}$ is to a scaled and permuted diagonal matrix, with an ISI of 0 representing a perfect source estimation, subject to scale and permutation ambiguities. The joint-ISI also penalizes when each $G^{\left[k\right]}$ has a different permutation structure, and thus, also accounts for the alignment of sources to SCVs within the JBSS estimation. We also measured the computational efficiency by measuring the wall time of the algorithms using the computational resources provided by the UMBC High Performance Computing Facility (HPCF); thus, our recorded wall times are reflective of the HPCF’s capabilities.

**Figure 2:**
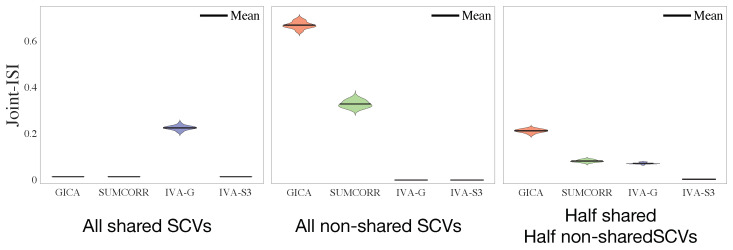
Joint-ISI performance of the four algorithms in the three different SCV simulation scenarios. The results are summarized from 20 independent simulations.

The joint-ISI performance is summarized in Figure 2. From the three scenarios, we observed that

- When all SCVs are shared, group-ICA and SUMCORR perform well, as their assumption that all SCVs are shared is a perfect model match with the simulated data. However, the IVA-G performs poorly because it heavily overparameterizes the SCVs (representing each effectively one-dimensional SCV by a K = 100 dimensional multivariate Gaussian). In contrast, the IVA-S3 provides a better estimation, as the SUMCORR initialization compensates for the shared SCVs.
- When all SCVs are non-shared, group-ICA and SUMCORR fail due to a complete model mismatch, whereas both the IVA-G and the IVA-S3 perform well because they are better suited to the estimation of more complex SCVs where greater variability exists across the datasets.
- When half of the SCVs are shared and half are non-shared, group-ICA and SUMCORR perform poorly, strictly because of the non-shared SCVs, whereas the IVA-G performs poorly, strictly because of the shared SCVs. In contrast, the IVA-S3 is able to estimate both shared and non-shared SCVs well. This is because the IVA-S3 leverages the strengths of both the SUMCORR and IVA by initializing the IVA with the SUMCORR estimation.

**Figure 3:**
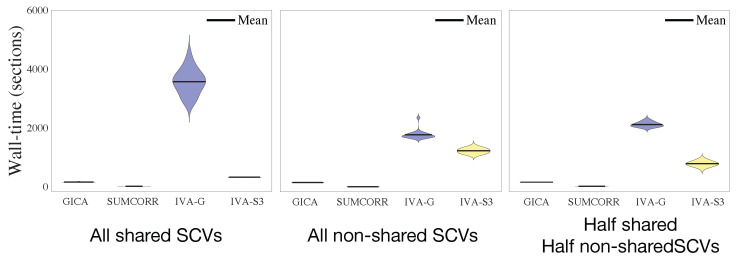
Wall times (s) of the four algorithms in the three different SCV simulation scenarios. The results are summarized from 20 independent simulations.

The wall times for all four algorithms are summarized in Figure 3. We first note that the IVA-G requires a greater wall time when sources are shared (and performance is worse). On the other hand, the IVA-S3 exhibits faster wall times than the IVA-G, the simplest and fastest IVA algorithm, owing, in part, to the good initialization of the IVA-G by the SUMCORR and, in part, due to the subspace separation afterwards. This reduction in computational time is especially valuable for applications such as fMRI data analysis, where many SCVs are shared; thus, the IVA-S3 is expected to provide an even greater time saving in the analysis of larger datasets.

### 5.2. Results with Real FMRI Data

Our application primarily involves neuroimaging data, specifically, fMRI data. Our simulated data experiments aimed to well-represent the statistics of real fMRI data. With the functional brain network components extracted from most JBSS methods, it is typically observed that a number of the source components are shared (are highly dependent) across all subjects, while a number of those are not [4,27]. The simulated SCVs (described above) correspond to such scenarios with both shared and non-shared fMRI sources. Although we simulated a scenario where half of the SCVs were shared and the other half were non-shared, it is crucial to recognize that real fMRI datasets are typically more complex and diverse in nature.

The data used in this study were resting-state fMRI data from the Bipolar–Schizophrenia Network on Intermediate Phenotypes (BSNIP) (https://nda.nih.gov/edit_collection.html?id=2274, accessed on 27 May 2023) [28,29,30,31]. We utilized data from a random subset of 49 healthy controls and 49 schizophrenia patients from the Baltimore site for this study. All participants were instructed to keep their eyes open, focus on a crosshair displayed on a monitor, and remain motionless during the entire scan, aided by a custom-built head-coil cushion that restricted head motion. Post-scan assessments were performed to confirm alertness. The imaging data were acquired using a 3-T scanner, and a single 5-min resting-state fMRI run was conducted, with a TR of 2.21. The following preprocessing steps were applied to the fMRI data: (i) rigid body motion correction to correct subject head motion; (ii) slice-timing correction to account for timing differences in slice acquisition; (iii) warping of the fMRI data into the standard Montreal Neurological Institute (MNI) [32] space using an echo-planar 412 imaging template and resampling to 3×3×3 mm^3^ isotropic voxels; and (iv) smoothing 413 of the resampled fMRI images using a Gaussian kernel with a full width at half maximum 414 (FWHM) of 6 mm. Each resting-state scan consisted of 134 volumes. To eliminate the 415 T1-related signal fluctuations (T1 effect) [33], the first three volumes were removed, leaving 131 volumes for each dataset used in this study. We performed masking on each volume to remove the non-brain voxels and flatten the result to form an observation vector of T = 63,304 voxels (samples).

Neither the MCCA nor the IVA need specific parameters for the algorithm, but the model order *N*, i.e., the dimension of the signal space, plays a crucial role. We estimated the model order based on information theoretic criteria. However, for fMRI data, the samples are not strictly independent and identically distributed samples (i.i.d samples); thus, classical order estimation techniques may overestimate the order. Downsampling is a common approach to combat this issue, but it can result in the loss of information. To overcome this limitation, two entropy rate (ER)-based order estimation techniques were used: ER with a finite memory length model and ER with an autoregressive model (AR) [34,35]. We examined the order with both methods, and the ER-FM yielded an order of 51±12, while the ER-AR yielded an order of 50±12. Using these values as guidance, we evaluated the consistency of group-ICA runs and the performance of IVA-G runs in multiple nearby orders to select the order that yielded the best performance, which ultimately led us to implement an order of N=55.

Due to the lack of ground truth sources for the real fMRI data, assessing the accuracy of the estimation is not possible, as it is for simulated cases where the true mixing is known. To evaluate the performance of our algorithm without a known ground truth, we first conducted a comparative analysis with the widely used group-ICA approach. We employed the back-reconstruction method “GICA” [16] in GIFT, which is the default in GIFT. Our evaluation was based on the identification of well-estimated components by both IVA-S3 and group-ICA methods in meaningful brain regions, such as the default-mode networks (DMN) and visual networks (VN), and this indicated reasonable performances by both algorithms. Unlike in the simulations, for these comparisons, we did not include results of the IVA-G or IVA-L-SOS, because the high number of fMRI datasets made the IVA algorithms computationally infeasible for this experiment.

**Figure 4:**
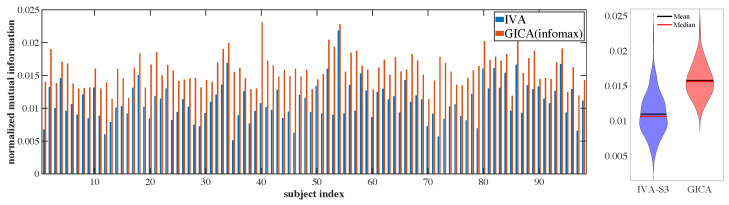
Normalized mutual information (MI) averaged across sources for each fMRI subject dataset (**left**), and violin plots of MI averaged over all 98 subjects (**right**).

Since JBSS algorithms typically separate sources through the minimization of mutual information, we also quantified the performance by measuring the mutual information (MI) between the estimated sources. Better separation of the sources would lead to smaller values for the mutual information between the estimated sources. We quantified the mutual information with the following measure [36]:

(10) $$I_{\mathrm{norm}}\left({\hat{y}}_{i},{\hat{y}}_{j}\right)=\frac{2I({\hat{y}}_{i},{\hat{y}}_{j})}{I\left({\hat{y}}_{i},{\hat{y}}_{i}\right)+I\left({\hat{y}}_{j},{\hat{y}}_{j}\right)}$$

where $I({\hat{y}}_{i},{\hat{y}}_{j})$ is the mutual information between two estimated components ${\hat{y}}_{i},{\hat{y}}_{j}$. For each dataset’s estimated sources $Y^{\left[k\right]}$, we calculated pairwise mutual information between all *N* sources within the dataset and averaged these N(N−1) pairwise MI values. We report the average MI value for each dataset in Figure 4 (left). In Figure 4 (right), we also include the average values across all datasets, representing the average level of dependence that each estimated source has to each other estimated source in its corresponding dataset. Here, we observed that the average normalized mutual information between estimated sources from the IVA-S3 was consistently lower than that obtained from group-ICA for all subjects, which indicates that the IVA-S3 could better separate the sources. The mean values of this normalized mutual information for all subjects were calculated and found to be 1.1 × $10^{-2}$ ± 2.9 × $10^{-3}$ for IVA-S3 and 1.6 × $10^{-2}$ ± 2.4 × $10^{-3}$ for group-ICA, which further supports this conclusion.

For more detailed assessments and comparisons of the performance of the two algorithms, we inspected all estimated components from both algorithms and selected those that appeared in both estimations and corresponded to each other. For most of these components, we observed that the estimates obtained by the IVA-S3 exhibited higher power ratios relative to those obtained by group-ICA, further indicating a better estimation quality for the IVA-S3. Among all matching components, the IVA-S3 estimations showed power ratios of 13.44±10.61 while those of group-ICA were 7.65±2.47.

**Figure 5:**
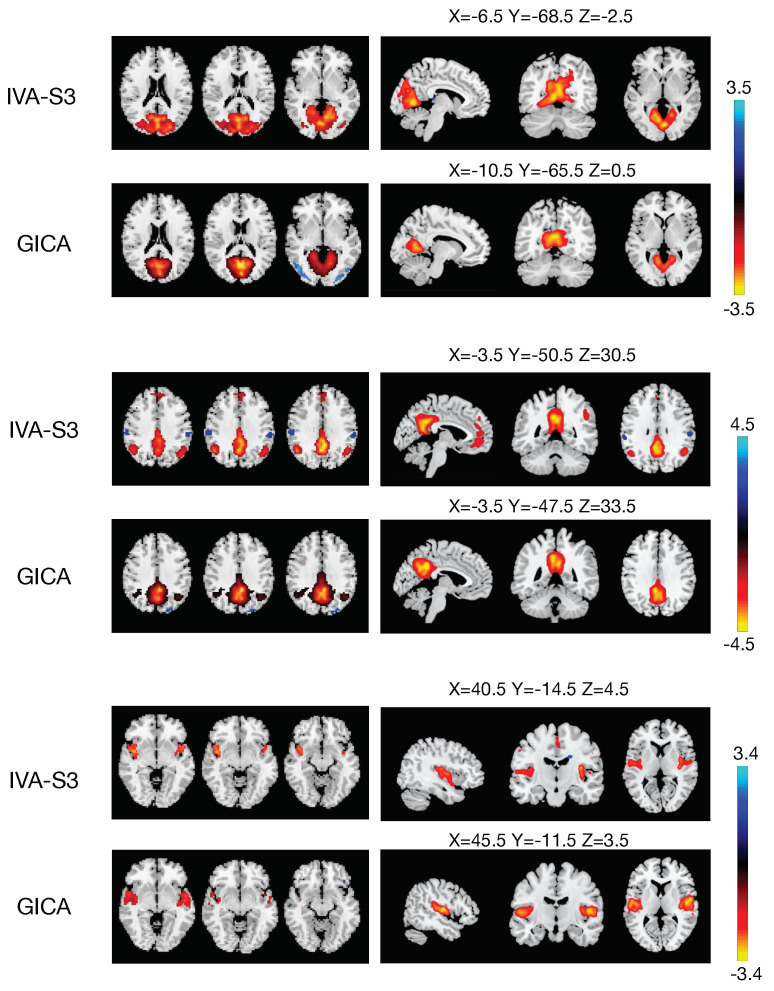
Example (one-sample) t-maps for components estimated from the IVA-S3 and group-ICA (Infomax), thresholded at p<0.05, after FDR correction. The IVA-S3 typically produces components with more activated voxels and better definitions of components, as is the case for the DMN.

Furthermore, we generated one-sample t-maps for the matching components and observed that the t-maps for the IVA-S3 estimations had more activated voxels in most components, indicating that the IVA-S3 preserved more subject variability. Examples of t-maps of matching components from different networks are presented in Figure 5.

We note that functional brain networks derived from fMRI data analysis play an important role in understanding mental disorders and brain function in general; thus, they are of great significance in medical research and neuroscience [30,37,38,39,40,41]. For example, schizophrenia is a complex mental disorder characterized by disordered thinking, emotional unresponsiveness, delusion, and hallucinations. The DMN is active during rest and self-referential thinking, and altered connectivity within the DMN is commonly observed in schizophrenia patients. Our results demonstrate that the IVA-S3 provides more precise definitions of all areas of the DMN, which contains multiple regions, such as the ventral anterior cingulate cortex (vACC), the posterior cingulate cortex (PCC), and the left and right inferior parietal cortexes (IPCs). Moreover, schizophrenia patients often suffer from impairments in visual perception, which are linked to brain regions such as the primary visual cortex and the lateral geniculate nucleus. Our results indicate that the IVA-S3 yields a better estimation of VN areas than the GICA, resulting in a greater number of activated voxels in one-sample t-maps and providing more detailed identification of visual area biomarkers. The precise estimations of functional brain networks allow for a better analysis of structural differences between healthy controls and schizophrenia patients, and the IVA-S3 offers a clear advantage over group-ICA in this regard.

## 6. Conclusions

In this study, we proposed and tested a scalable IVA algorithm by first identifying and separating the “shared” subspace from the data. Our method combines the strengths of both the SUMCORR and IVA-G by initializing the IVA-G with SUMCORR estimation, providing an estimation procedure that is well-suited for estimating the shared sources, thus helping us to identify and separate the shared and non-shared subspaces effectively. By reducing the dimensionality of the problem, our approach also enables the IVA to be conducted over larger numbers of datasets. After testing our method on both simulated datasets and real fMRI datasets, our results demonstrate that the IVA-S3 inherits the robust estimation performance of the IVA-G while also being able to estimate low-rank shared subspaces in a superior manner to the use of the IVA-G alone, in addition to providing improved scalability to higher numbers of SCVs than the IVA-G alone. Our findings indicate the IVA-S3 offers the potential for improved subgroup identification with fMRI data, as we demonstrated that the IVA-S3 is able to better preserve subject variability. A more detailed investigation of subgroup identification is beyond the scope of this work but is an emerging new area of research [4,42,43,44,45]. Furthermore, as discussed in the previous section, the IVA might be prone to overfitting when analyzing large numbers of datasets. In addition to the separation of subspaces, it might be desirable to have methods of effective quantitative analysis of the datasets themselves prior to the analysis to assess the suitability of a set of datasets for IVA analysis and its evaluation in terms of the complexity of the models employed. In summary, the IVA-S3 can achieve excellent estimation performances with significantly reduced computational costs, making it a promising method for large-scale fMRI analysis.

## Data Availability

The datasets used in this study are publicly available at https://nda.nih.gov/edit_collection.html?id=2274, accessed on 27 May 2023.

## Abbreviations

The following abbreviations are used in this manuscript:

BSS	Blind source separation
JBSS	Joint blind source separation
fMRI	Functional magnetic resonance imaging
CCA	Canonical correlation analysis
CVs	Canonical variate
MCCA	Multi-set canonical correlation analysis
SUMCORR	Maximizing the sum of correlations
ICA	Independent component analysis
Group-ICA	Group independent component analysis
IVA	Independent vector analysis
IVA-G	Independent vector analysis with a multivariate Gaussian source prior
IVA-L-SOS	Independent vector analysis with a multivariate Laplacian distribution and second-order statistics
SCV	Source component vector
PCA	Principal component analysis
PC	Principal component
DMN	Default-mode networks
VN	Visual networks
MGGD	Multivariate generalized Gaussian distributed
IVA-S3	Independent vector analysis
